# Prevalence of Gestational Diabetes Mellitus in Eastern and Southeastern Asia: A Systematic Review and Meta-Analysis

**DOI:** 10.1155/2018/6536974

**Published:** 2018-02-20

**Authors:** Cong Luat Nguyen, Ngoc Minh Pham, Colin W. Binns, Dat Van Duong, Andy H. Lee

**Affiliations:** ^1^School of Public Health, Curtin University, Perth, WA, Australia; ^2^National Institute of Hygiene and Epidemiology, Hanoi, Vietnam; ^3^Thai Nguyen University of Medicine and Pharmacy, Thai Nguyen, Vietnam; ^4^United Nations Population Fund, Hanoi, Vietnam

## Abstract

**Aim:**

To review the prevalence of gestational diabetes mellitus (GDM) in Eastern and Southeastern Asia.

**Methods:**

We systematically searched for observational studies on GDM prevalence from January 2000 to December 2016. Inclusion criteria were original English papers, with full texts published in peer-reviewed journals. The quality of included studies was evaluated using the guidelines of the National Health and Medical Research Council, Australia. Fixed effects and random effects models were used to estimate the summary prevalence of GDM and the corresponding 95% confidence intervals (CI).

**Results:**

A total of 4415 papers were screened, and 48 studies with 63 GDM prevalence observations were included in the final review. The pooled prevalence of GDM was 10.1% (95% CI: 6.5%–15.7%), despite substantial variations across nations. The prevalence of GDM in lower- or upper-middle income countries was about 64% higher than in their high-income counterparts. Moreover, the one-step screening method was twice more likely to be used in diagnosing GDM when compared to the two-step screening procedure.

**Conclusions:**

The prevalence of GDM in Eastern and Southeastern Asia was high and varied among and within countries. There is a need for international uniformity in screening strategies and diagnostic criteria for GDM.

## 1. Introduction

Gestational diabetes mellitus (GDM), which is defined as diabetes diagnosed in the second and third trimesters of pregnancy [1], has emerged as a global public health concern [2]. It has been associated with short-term and long-term adverse health outcomes for both mothers and their newborns [3]. Women with GDM are known to have decreased quality of life and increased risks of caesarean section, gestational hypertension, preeclampsia, and type 2 diabetes [4–7]. In babies, GDM has been found to be associated with macrosomia or larger than normal gestational-aged infants, neonatal hypoglycemia, and type 2 diabetes mellitus later in life [6, 8, 9]. As such, it is important to understand the burden of GDM in various parts of the world to provide country-specific information to help inform on policy and planning.

The global prevalence of GDM varies widely, from 1% to 28%, depending on population characteristics (e.g., maternal age, socioeconomic status, race/ethnicity, or body composition), screening methods, and diagnostic criteria [10]. In addition, as with the common form of type 2 diabetes [11], GDM can also be influenced by genetic factors, which may differently affect disease prevalence among populations [12]. To date, at least 8 associations have developed their own diagnostic criteria for GDM, namely, the American Diabetes Association (ADA 2004, 2007, 2010, and 2012), Australian Diabetes in Pregnancy Society (ADIPS), Carpenter-Coustan (CC), International Association of the Diabetes and Pregnancy Study Groups (IADPSG), International Classification of Diseases (ICD), Japan Society of Obstetrics and Gynecology (JSOG), National Diabetes Data Group (NDDG), and World Health Organization (WHO 1998, 1999, 2006, and 2013) [13, 14]. Data in high-income countries (HICs) ranges from 0.6% to 27.5% [15], and those in low- and middle-income countries are in the range of 0.4 and 24.3% [16]. Regional differences exist regarding the distribution of GDM, such as Africa and Asia, after adjusting the data with prevalence reports being 0%–13.9% and 1.6%–17.8%, respectively [17, 18].

Asia is the largest and most populated continent (60% of the world's population), with an increasing prevalence of GDM [19]. While maternal overweight/obesity is an established risk factor for GDM [20], particularly in HICs, recent reviews have found that the prevalence of GDM may be even higher among lean populations than those with a larger body size [2]. This is consistent with the developmental origins of adult disease hypothesis (DOHAD) as undernutrition in the first 1000 days is associated with later diabetes [21–24]. The Eastern and Southeastern subregions include 18 countries, with more than 30% of the Asian population [25] and contributing approximately 80% to the Asian economy [26]. Given the rapid socioeconomic and nutrition transition and the increasing prevalence of GDM in Asia [19, 27], it is of public health importance to provide an overview of this condition in Eastern and Southeastern Asia. However, accessible and systematically organized estimates of GDM prevalence in this subregion are lacking. Moreover, the lack of uniformity in screening methods, definition, and diagnostic criteria for GDM makes it difficult to compare the prevalence of GDM between and within countries. The aim of this study was to undertake a systematic review and meta-analysis of the prevalence and associated risk factors of GDM in selected countries of Eastern and Southeastern Asia.

## 2. Methods

The present review was conducted according to the Preferred Reporting Items for Systematic Reviews and Meta-Analyses (PRISMA) statement [28] and the Meta-analysis Of Observational Studies in Epidemiology (MOOSE) protocols [29].

### 2.1. Search Strategy

The databases (PubMed, Embase, and Scopus) were used to search relevant articles with the following key words: “gestational diabetes mellitus,” “GDM,” “hyperglycemia in pregnancy,” “gestational hyperglycemia,” or “diabetes in pregnancy” as well as “name of country” in Eastern and Southeastern Asia. The websites of the World Health Organization (WHO) and International Diabetes Federation were also reviewed to extend our search results. Then the reference lists of included articles were assessed to identify further relevant studies.

### 2.2. Inclusion Criteria

Studies that met the following criteria were retrieved for assessment: (1) being conducted in Eastern and Southeastern Asian countries classified by the United Nations Statistics Division [30]; (2) being published in English language journals between January 1, 2000, and December 31, 2016; (3) reported primary results (i.e., original studies); (4) provided the prevalence of GDM and associated 95% confidence interval (CI) or total of participants and number of GDM events; and (5) had a sample size of at least 1000 and 50 GDM cases. When multiple publications were derived from analyses of the same or overlapping samples, we used data from the largest or most recent results only.

### 2.3. Study Selection

Relevant papers identified from the aforementioned databases and websites were imported into an EndNote X7.5, and duplicates were removed. Two reviewers independently screened the titles and abstracts for potentially eligible articles based on the inclusion criteria. If a paper contained insufficient information on GDM in the title and/or abstract, the full text was retrieved for further assessment and any disagreement between the two reviewers was resolved through discussion. Finally, the full text of relevant studies was reviewed.

### 2.4. Quality Assessment and Data Extraction

The guidelines of the National Health and Medical Research Council were used to assess the quality of searched articles by two independent investigators [31]. Only articles that meet the level III of evidence were included and analysed in this review. An extraction form was developed in Excel to record data from selected papers by one reviewer, and the completeness and accuracy of extracted data were verified by a second reviewer. The following characteristics were extracted from each study: first author, country, year of publication, year of survey, setting, gestational age, screening procedure (one and/or two steps), sample size, GDM cases, prevalence of GDM (including percentage and 95% CI), and diagnostic criteria for GDM. If 95% CIs were not reported, they were calculated based on the sample size and observed proportion of GDM in each selected study [32]. Since we only collected published studies, ethical approval was not required for this work.

### 2.5. Data Analysis

Diagnostic criteria were aggregated into 8 clusters due to some similarities: (1) JSOG, (2) NDDG, (3) ADA 2004/ADA 2010, (4) ICD10, (5) ADA 2007/CC, (6) WHO 1998–2006, (7) ADA 2012/IADPSG/WHO 2013; and (8) ADIPS98. The prevalences of GDM, with 95% CI, were grouped according to the different diagnostic criteria to perform meta-analyses. The summary prevalence (95% CI) regardless of and by each diagnostic criteria was calculated using the random effects model of the DerSimonian and Laird method [33] to allow for the possibility of real differences in the distribution of GDM between studies that are not solely resulted from sampling error. The heterogeneity among studies was tested with the *I* ^2^ index (low is <25%, moderate 25%–50%, and high > 50%), which describes the percent of total variation contributed by between-study variations [34]. The overall prevalence of GDM (95% CI) by each group of diagnostic criteria was depicted graphically in forest plots. Statistically significant heterogeneity was considered present at *P* < 0.1 and *I* ^2^ > 50% [35]. In addition, subgroup analysis according to lower- or upper-middle income countries (LMICs) or HICs, type of GDM screening, and individual country under study was performed to understand the impact of economic development and geographical location on the prevalence of GDM. The summary prevalence of GDM for each study that used more than one diagnostic criterion was pooled using a fixed effects model. All analyses were performed using Stata 13.1 (StataCorp LP, College Station, TX).

## 3. Results

### 3.1. Description of Included Studies

**Figure 1:**
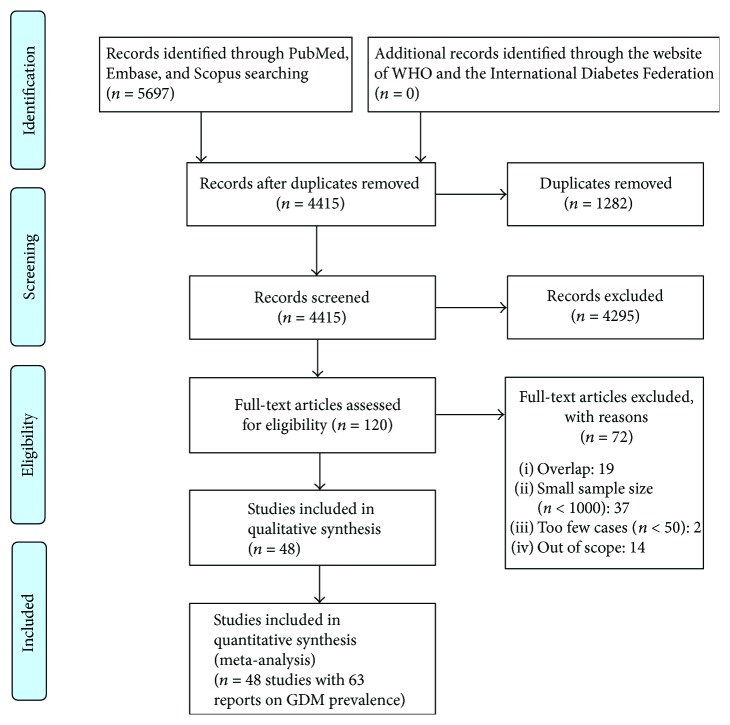
PRISMA flow diagram of selected studies.

Figure 1 shows the flow diagram of our systematic literature search. The initial search identified 5697 publications, and after the removal of duplicate records (*n* = 1282), 4415 were retrieved for preliminary assessment. Of these, 120 were potentially relevant after title and abstract screening, and thus, their full texts were obtained and evaluated against the inclusion criteria, resulting in 48 studies reported in 63 observations. No papers were retrieved from the reference list. Of 48 studies, one paper reported four values of GDM prevalence by using four diagnostic criteria [36], 12 papers had two values of GDM prevalence by comparing two different diagnostic criteria or screening types [37–48], and 35 papers only used one diagnostic criterion to estimate GDM prevalence [49–83].

### 3.2. Characteristics of Included Studies

The main characteristics of the included studies are described in the Supplemental Table (available here). Between the years 2000 and 2016, 48 articles were published with a total sample of 3,594,803 pregnant women (range: 1038–1,824,913) in 7 countries. Of the 48 studies, 21 were conducted in China [41–43, 45, 48–64], 8 in the Republic of Korea [65, 68, 70–72, 74, 75, 77], 6 in Thailand [37, 46, 79–81, 83], five in Japan [39, 47, 73, 76, 78], five in Taiwan (China) [38, 44, 66, 67, 69], one in Malaysia [82], one in Singapore [40], and one in Vietnam [36]. Two-thirds of the studies (*n* = 32) used a two-step screening procedure, that is, women underwent a 1-hour glucose challenge test (GCT) and a 3-hour glucose tolerance test (GTT) if GCT were abnormal. To perform these tests, women were required to drink 50 g of glucose and 75 or 100 g of glucose for GCT and GTT, respectively. Over one-quarter of studies (*n* = 13) followed a single-step screening, where all pregnant women were given a 75 g GTT. Three studies did not specify the screening method used [56, 71, 75]. A total of 20 studies used IADPSG, the 2010 ADA, or the 2013 WHO standards as the GDM diagnostic criteria. The number of studies that employed CC or the 2007 ADA, NDDG, WHO (1998, 1999, and 2006) was 13, 12, and 10, respectively. The remaining 8 studies applied other criteria (see Supplemental Table). All studies included in the present review met the level III of evidence of the National Health and Medical Research Council in Australia [31].

### 3.3. Prevalence of GDM

**Figure 2:**
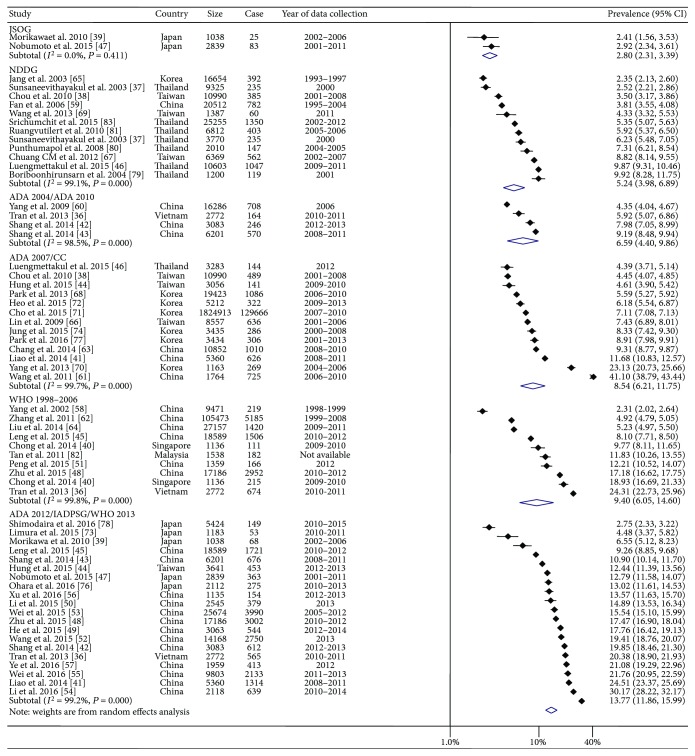
Forest plots presenting the prevalence of gestational diabetes for individual studies and the corresponding pooled prevalence for studies combined according to diagnostic criteria in Eastern and Southeastern Asia. Bars and diamonds indicate 95% confidence interval (CI). The size of each square corresponds to the weight of the study in the meta-analysis using the Der Simonian and Laird method. ADA: American Diabetes Association; CC: Carpenter-Coustan; IADPSG: International Association of the Diabetes and Pregnancy Study Groups; JSOG: Japan Society of Obstetrics and Gynecology; NDDG: National Diabetes Data Group; WHO: World Health Organization.

The overall mean prevalence of GDM, regardless of diagnostic standards, was 10.07 (95% CI: 6.47–15.68) (Table 1).  Figure 2 depicts the prevalence of GDM across 8 diagnostic groupings. The highest prevalence of GDM was observed for studies using the IADPSG, ADA 2012, or WHO 2013 criteria (13.77%) while the lowest data was found among Japanese reports that employed JSOG criteria (2.80%). Between that range, the summary prevalence of GDM according to NDDG, ADA 2004 or ADA 2010, ADA 2007 or CC, and WHO (1998, 1999, or 2006) recommendations was 5.24%, 6.59%, 8.54%, and 9.40%, respectively. Only two single data points for GDM prevalence were reported using either ICD 10 [75] or ADIPS 1998 [36], with the respective prevalences being 7.53% and 20.82%, respectively (Table 1). With the exception of studies employing JSOG, there was considerable heterogeneity of GDM prevalence among studies assessed based on various criteria; a measure of heterogeneity varied from 98.5% to 99.8% (*P* < 0.001).

### 3.4. Prevalence of GDM by Income and Diagnostic Criteria

Overall, the prevalence of GDM was higher in LMICs than HICs, 10.84% versus 6.66%, respectively (Table 1). Except for pooled GDM prevalence according to WHO (1998, 1999, or 2006) criteria, the summary estimates of GDM prevalence based on other diagnostic criteria were greater in LMICs than in HICs. Notably, the prevalence using the most popular criteria, that is, ADA 2012, IADPSG, or WHO 2013, was over twofold higher in the former when compared with the corresponding figure in the latter (17.56% versus 7.48%) (Table 1).

### 3.5. Prevalence of GDM by Screening Method

The mean prevalence of GDM derived using one-step screening and two-step screening was 15.71% (95% CI: 13.88–17.77%) and 7.15% (95%CI: 5.63–9.08%), respectively; there was substantial heterogeneity among studies using either the one-step screening method or the two-step screening method (*I* ^2^ > 98% and *P* < 0.001) (Table 1).

### 3.6. Prevalence of GDM by Country

There was variation in the overall prevalence of GDM, with Vietnam and Singapore showing the highest rates (20.06% and 18.93%, resp.). While mainland China and Malaysia had a comparable prevalence of GDM (11.91% and 11.83%), the remaining countries (Japan, Korea, Taiwan, and Thailand) had a GDM prevalence of less than 8.0%. It should be noted that mainland China accounted for nearly 50% of the total studies (*n* = 21) (Table 1).

## 4. Discussion

The present review included 48 studies with more than three and a half million participants from 7 countries in Eastern and Southeastern Asia, showing a wide variation in the overall prevalence of GDM. The pooled prevalence of GDM was approximately 10%, with a higher estimate in LMICs than in HICs. The discrepancy in the overall estimate also existed according to diagnostic criteria and countries. The most widely used criteria were ADA 2012, IADPSG, or WHO 2013, resulting in a pooled prevalence of GDM of 14% while only a limited number of studies used ADIP 1998, ICD 10, JSOG, ADA 2004, or ADA 2010. The highest prevalence of GDM was found in Vietnam and Singapore, where approximately one in five women were diagnosed with GDM, followed by mainland China and Malaysia where about one in 9 women had GDM. The remaining countries had no more than one in 14 women with GDM. To the best of our knowledge, this is the first study that systematically synthesised data on the prevalence of GDM in important subregions of Asia, Eastern and Southeastern, and provided accessible evidence to formulate locally feasible strategies for effective and efficient prevention of GDM in Asia.

Overall, approximately one in 10 pregnant women in Eastern and Southeastern Asia had GDM. This finding is higher than African countries, where the average prevalence of GDM is about 6.0% [17]. Similarly, our data is greater than results reported in Western countries including Europe, US, and Australia, with the prevalence of GDM being 5.4%, 9.2%, and 5.7%, respectively [84–86]. We have no clear reason for such a discrepancy, but we speculate that it may be due to socioeconomic, racial/ethnic, or lifestyle disparities. For instance, Asian women were reportedly having a higher risk of GDM compared with their Caucasian, African-American, and Hispanic counterparts [87]. This observation suggests that the development of GDM may be shaped by early-life exposure to poor nutrition, that is, under- or overnutrition, and/or epigenetics according to the DOHAD theory [88]. Another factor may be the different screening regimes and testing methods that will be discussed below.

The lack of consensus regarding the use of diagnostic criteria for GDM is largely attributable to the heterogeneity of GDM prevalence. Of diverse diagnostic criteria such as NDDG [89], CC [90], ADA [91], and WHO [92], the IADPSG criteria based on the Hyperglycemia and Adverse Pregnancy Outcome Study (HAPO) has recently become more accepted [93]. Indeed, the use of IADPSG criteria may produce an estimated prevalence of GDM two to threefold even up to 7-fold higher than other criteria [13, 94]. In a Brazilian study, for instance, the prevalence of GDM was only 2.3% and 7.1% according to ADA 2010 and WHO 1999, respectively, but it increased to 18.0% following the IADPSG criteria [94]. An alternative explanation for the variation in GDM prevalence may be ascribed to different screening methods, that is, the one-step or two-step approach. Similar to our review, a recent meta-analysis of 40 studies in Europe reported that the one-step screening method resulted in a higher prevalence of GDM compared with the two-step procedure [86]. Although a one-step screening type is simpler, less laborious, and of low lost, it typically overestimates the prevalence of GDM [95]. However a two-step screening method is more accurate and could accordingly reduce personal and societal costs despite its inconvenience for patients and increased workload for healthcare professionals [96]. Given the lack of international consensus in screening and diagnostic methods for GDM, it is imperative to develop a standardised approach to allow for comparison of GDM burdens worldwide.

The high prevalences of GDM in less wealthy countries reviewed here are consistent with studies from other parts of Asia and Africa [17, 18]. Likewise, around 90% of cases of hyperglycemia during pregnancy occur in low- and middle-income nations as reported by the International Diabetes Federation in 2015 [27]. This discrepancy may be associated with limited access to maternal health care and/or low socioeconomic status in low- and middle-income economies [27, 97, 98]. It is evident from this review that the prevalence of GDM in Vietnam, a lower-middle income country, at least tripled the corresponding data in HICs such as Japan, Taiwan, and South Korea. It can also be speculated that the difference in lifestyle factors (e.g., diet and physical activity), acculturation, and urbanisation may explain the variation in GDM prevalence between the two aforementioned country-income groups [99]. This finding implies that improvement of socioeconomic conditions may contribute to the prevention of GDM.

On the other hand, more epidemiological studies on GDM in the remaining countries of Eastern and Southeastern Asian regions including Mongolia, Indonesia, Philippines, Myanmar, Cambodia, and Laos need to be conducted to add information to the current evidence. These studies should be performed in both urban and rural populations in order to compare and evaluate the effects of urbanisation on GDM in particular and public health in general.

The present review has the advantages of a large sample size with studies involving over three and a half million women, using different methods for screening and diagnosis of GDM and consistency of method, quality, and focus. There are several limitations that need to be considered when interpreting the results of this work. Our review indicated substantial heterogeneity of GDM prevalence across studies, making direct comparison difficult. Such variation may be attributable to the potential influence of screening procedures (i.e., selective or universal) for GDM and its diagnostic criteria, population characteristics, or other socioenvironmental factors. Nonetheless, those possible modifiers were not taken into account in this review due to the lack of data available from included studies. In addition, the inclusion of only English publications may have resulted in publication bias. Our review did not address GDM situations in other countries in the region including Indonesia, Philippines, Myanmar, Cambodia, and Laos due to the lack of data, and thus, the findings may not be generalisable to the whole Eastern and Southeastern Asia.

## 5. Conclusion

A large-scale review of literature shows that around one in 10 pregnant women in Eastern and Southeastern Asia had GDM and the number of women with GDM varied substantially between and within countries. The prevalence of GDM was highest according to ADA 2012, IADPSG, or WHO 2013 criteria, greater following a one-step screening procedure and higher in LMICs. The findings suggest the need for developing an international uniformity regarding screening and diagnostic methods for GDM.

## Figures and Tables

**Table 1:** Pooled prevalence and 95% confidence interval of gestational diabetes according to the income group, screening type, and country.

	Studies	Subjects	Prevalence	Lower 95% CI	Upper 95% CI	*I* ^2^	*P*_heterogeneity_
Income level							
High							
JSOG	2	3877	2.80	2.31	3.39	0.00%	0.411
NDDG	4	35,400	4.21	2.15	8.26	99.30%	<0.001
ICD10	1	1,306,281	7.53	7.51	7.56	—	—
ADA 2007/CC	9	1,880,183	7.38	6.03	9.03	98.90%	<0.001
WHO 1999–2006	2	2272	15.37	13.89	17.02	—	—
ADA 2012/IADPSG/WHO 2013	6	16,237	7.48	4.74	11.80	98.60%	<0.001
Subtotal	24	3,244,250	6.66	4.40	10.09	98.30%	<0.001
Lower- or upper-middle							
NDDG	8	79,487	5.83	4.31	7.90	99.10%	<0.001
ADA 2004/ADA 2010	4	28,342	6.59	4.40	9.86	98.50%	<0.001
ADA 2007/CC	4	21,259	11.85	4.94	28.42	99.80%	<0.001
WHO 1999–2006	8	183,545	8.57	5.23	14.06	99.90%	<0.001
ADA 2012/IADPSG/WHO 2013	14	113,656	17.56	15.07	20.47	99.20%	<0.001
ADIPS98	1	2772	20.82	19.34	22.40	—	—
Subtotal	39	429,061	10.84	7.35	15.99	94.40%	<0.001
Type of screening							
One step	13	95,638	15.71	13.88	17.77	98.90%	<0.001
Two-step	32	338,825	7.15	5.63	9.08	99.70%	<0.001
Unspecified	3	3,132,329	7.83	7.39	8.29	99.70%	<0.001
Country							
Mainland China	21	282,086	11.91	8.96	15.83	99.90%	<0.001
Japan	5	12,596	6.08	3.49	10.62	98.70%	<0.001
Korea	8	3,180,515	7.12	6.74	7.53	99.60%	<0.001
Malaysia	1	1538	11.83	10.30	13.60	—	—
Singapore	1	1136	18.93	16.74	21.40	—	—
Taiwan	5	30,944	6.51	4.45	9.54	99.0%	<0.001
Thailand	6	55,205	6.10	4.39	8.48	98.8%	<0.001
Vietnam	1	2772	20.06	19.28	20.87	—	—
All	48	3,566,792	10.07	6.47	15.68	99.3%	<0.001

—: not applicable; ADA: American Diabetes Association; ADIPS: Australian Diabetes in Pregnancy Society; CC: Carpenter-Coustan; IADPSG: International Association of the Diabetes and Pregnancy Study Groups; ICD: International Classification of Diseases; JSOG: Japan Society of Obstetrics and Gynecology; NDDG: National Diabetes Data Group; WHO: World Health Organization.

## Supplementary Materials

Supplementary Materials Supplemental Table: characteristics of selected studies.

## References

[1] American Diabetes Association (2015). 2. Classification and diagnosis of diabetes. *Diabetes Care*.

[2] Guariguata L., Linnenkamp U., Beagley J., Whiting D. R., Cho N. H. (2014). Global estimates of the prevalence of hyperglycaemia in pregnancy. *Diabetes Research and Clinical Practice*.

[3] Farrar D., Simmonds M., Bryant M. (2016). Hyperglycaemia and risk of adverse perinatal outcomes: systematic review and meta-analysis. *BMJ*.

[4] Kim C., Newton K. M., Knopp R. H. (2002). Gestational diabetes and the incidence of type 2 diabetes: a systematic review. *Diabetes Care*.

[5] Yogev Y., Xenakis E. M. J., Langer O. (2004). The association between preeclampsia and the severity of gestational diabetes: the impact of glycemic control. *American Journal of Obstetrics and Gynecology*.

[6] HAPO Study Cooperative Research Group, Metzger B. E., Lowe L. P. (2008). Hyperglycemia and adverse pregnancy outcomes. *The New England Journal of Medicine*.

[7] Marchetti D., Carrozzino D., Fraticelli F., Fulcheri M., Vitacolonna E. (2017). Quality of life in women with gestational diabetes mellitus: a systematic review. *Journal of Diabetes Research*.

[8] Langer O., Yogev Y., Most O., Xenakis E. M. J. (2005). Gestational diabetes: the consequences of not treating. *American Journal of Obstetrics and Gynecology*.

[9] Clausen T. D., Mathiesen E. R., Hansen T. (2008). High prevalence of type 2 diabetes and pre-diabetes in adult offspring of women with gestational diabetes mellitus or type 1 diabetes. *Diabetes Care*.

[10] Jiwani A., Marseille E., Lohse N., Damm P., Hod M., Kahn J. G. (2012). Gestational diabetes mellitus: results from a survey of country prevalence and practices. *The Journal of Maternal-Fetal & Neonatal Medicine*.

[11] Pullinger C. R., Goldfine I. D., Tanyolaç S. (2014). Evidence that an *HMGA1* gene variant associates with type 2 diabetes, body mass index, and high-density lipoprotein cholesterol in a Hispanic-American population. *Metabolic Syndrome and Related Disorders*.

[12] Yan J., Su R., Ao D., Wang Y., Wang H., Yang H. (2017). Genetic variants and clinical relevance associated with gestational diabetes mellitus in Chinese women: a case-control study. *The Journal of Maternal-Fetal & Neonatal Medicine*.

[13] Agarwal M. M. (2015). Gestational diabetes mellitus: an update on the current international diagnostic criteria. *World Journal of Diabetes*.

[14] Mack L. R., Tomich P. G. (2017). Gestational diabetes: diagnosis, classification, and clinical care. *Obstetrics and Gynecology Clinics of North America*.

[15] Chiefari E., Arcidiacono B., Foti D., Brunetti A. (2017). Gestational diabetes mellitus: an updated overview. *Journal of Endocrinological Investigation*.

[16] Kanguru L., Bezawada N., Hussein J., Bell J. (2014). The burden of diabetes mellitus during pregnancy in low- and middle-income countries: a systematic review. *Global Health Action*.

[17] Macaulay S., Dunger D. B., Norris S. A. (2014). Gestational diabetes mellitus in Africa: a systematic review. *PLoS One*.

[18] Hirst J., Raynes-Greenow C., Jeffery H. (2012). A systematic review of trends of gestational diabetes mellitus in Asia. *Journal of Diabetology*.

[19] Tutino G. E., Tam W. H., Yang X., Chan J. C. N., Lao T. T. H., Ma R. C. W. (2014). Diabetes and pregnancy: perspectives from Asia. *Diabetic Medicine*.

[20] Chu S. Y., Callaghan W. M., Kim S. Y. (2007). Maternal obesity and risk of gestational diabetes mellitus. *Diabetes Care*.

[21] Vaiserman A. (2017). Early-life nutritional programming of type 2 diabetes: experimental and quasi-experimental evidence. *Nutrients*.

[22] Wong I. O. L., Cowling B. J., Schooling C. M. (2015). Vulnerability to diabetes in Chinese: an age–period–cohort analysis. *Annals of Epidemiology*.

[23] Jiang X., Ma H., Wang Y., Liu Y. (2013). Early life factors and type 2 diabetes mellitus. *Journal of Diabetes Research*.

[24] Krishnaveni G. V., Yajnik C. S. (2017). Developmental origins of diabetes—an Indian perspective. *European Journal of Clinical Nutrition*.

[25] The United Nations Demographic yearbook 2015. https://unstats.un.org/unsd/demographic/products/dyb/dyb2015/Table01.pdf.

[26] International Monetary Fund World economic outlook database. https://www.imf.org/external/pubs/ft/weo/2017/01/weodata/index.aspx.

[27] International Diabetes Federation (2015). *IDF Diabetes Atlas*.

[28] Moher D., Liberati A., Tetzlaff J., Altman D. G., PRISMA Group (2010). Preferred reporting items for systematic reviews and meta-analyses: the PRISMA statement. *International Journal of Surgery*.

[29] Stroup D. F., Berlin J. A., Morton S. C. (2000). Meta-analysis of observational studies in epidemiology: a proposal for reporting. *JAMA*.

[30] United Nations Statistics Division Geographical region and composition of each region. http://unstats.un.org/unsd/methods/m49/m49regin.htm#asia.

[31] National Health and Medical Research Council NHMRC additional levels of evidence and grades for recommendations for developers of guidelines. https://www.nhmrc.gov.au/_files_nhmrc/file/guidelines/developers/nhmrc_levels_grades_evidence_120423.pdf.

[32] Gardner M. J., Altman D. G. (1986). Confidence intervals rather than P values: estimation rather than hypothesis testing. *BMJ*.

[33] DerSimonian R., Laird N. (1986). Meta-analysis in clinical trials. *Controlled Clinical Trials*.

[34] Higgins J. P., Thompson S. G. (2011). *Cochrane Handbook for Systematic Reviews of Interventions Version 5.1.0 (Updated March 2011)*.

[35] Higgins J. P. T., Thompson S. G. (2002). Quantifying heterogeneity in a meta-analysis. *Statistics in Medicine*.

[36] Tran T. S., Hirst J. E., Do M. A. T., Morris J. M., Jeffery H. E. (2013). Early prediction of gestational diabetes mellitus in Vietnam: clinical impact of currently recommended diagnostic criteria. *Diabetes Care*.

[37] Sunsaneevithayakul P., Boriboohirunsarn D., Sutanthavibul A. (2003). Risk factor-based selective screening program for gestational diabetes mellitus in Siriraj Hospital: result from clinical practice guideline. *Journal of the Medical Association of Thailand*.

[38] Chou C. Y., Lin C. L., Yang C. K., Yang W. C., Lee F. K., Tsai M. S. (2010). Pregnancy outcomes of Taiwanese women with gestational diabetes mellitus: a comparison of Carpenter-Coustan and National Diabetes Data Group Criteria. *Journal of Women's Health*.

[39] Morikawa M., Yamada T., Yamada T. (2010). Change in the number of patients after the adoption of IADPSG criteria for hyperglycemia during pregnancy in Japanese women. *Diabetes Research and Clinical Practice*.

[40] Chong Y.-S., Cai S., Lin H. (2014). Ethnic differences translate to inadequacy of high-risk screening for gestational diabetes mellitus in an Asian population: a cohort study. *BMC Pregnancy and Childbirth*.

[41] Liao S., Mei J., Song W. (2014). The impact of the International Association of Diabetes and Pregnancy Study Groups (IADPSG) fasting glucose diagnostic criterion on the prevalence and outcomes of gestational diabetes mellitus in Han Chinese women. *Diabetic Medicine*.

[42] Shang M., Lin L. (2014). IADPSG criteria for diagnosing gestational diabetes mellitus and predicting adverse pregnancy outcomes. *Journal of Perinatology*.

[43] Shang M., Lin L., Ma L., Yin L. (2014). Investigation on the suitability of the International Association of Diabetes and Pregnancy Study Group diagnostic criteria for gestational diabetes mellitus in China. *Journal of Obstetrics and Gynaecology*.

[44] Hung T. H., Hsieh T. T. (2015). The effects of implementing the International Association of Diabetes and Pregnancy Study Groups Criteria for diagnosing gestational diabetes on maternal and neonatal outcomes. *PLoS One*.

[45] Leng J., Shao P., Zhang C. (2015). Prevalence of gestational diabetes mellitus and its risk factors in Chinese pregnant women: a prospective population-based study in Tianjin, China. *PLoS One*.

[46] Luengmettakul J., Sunsaneevithayakul P., Talungchit P. (2015). Pregnancy outcome in women with gestational diabetes mellitus according to the Carpenter–Coustan criteria in Thailand. *The Journal of Obstetrics and Gynaecology Research*.

[47] Nobumoto E., Masuyama H., Hiramatsu Y., Sugiyama T., Kusaka H., Toyoda N. (2015). Effect of the new diagnostic criteria for gestational diabetes mellitus among Japanese women. *Diabetology International*.

[48] Yang H., Zhang M., Zhang H. (2015). Comparing the diagnostic criteria for gestational diabetes mellitus of World Health Organization 2013 with 1999 in Chinese population. *Chinese Medical Journal*.

[49] He J. R., Yuan M. Y., Chen N. N. (2015). Maternal dietary patterns and gestational diabetes mellitus: a large prospective cohort study in China. *British Journal of Nutrition*.

[50] Li G., Kong L., Zhang L. (2015). Early pregnancy maternal lipid profiles and the risk of gestational diabetes mellitus stratified for body mass index. *Reproductive Sciences*.

[51] Peng S., Liu L., Zhang X. (2015). A nested case-control study indicating heavy metal residues in meconium associate with maternal gestational diabetes mellitus risk. *Environmental Health*.

[52] Wang C., Zhu W., Wei Y., Feng H., Su R., Yang H. (2015). Exercise intervention during pregnancy can be used to manage weight gain and improve pregnancy outcomes in women with gestational diabetes mellitus. *BMC Pregnancy and Childbirth*.

[53] Wei Y. M., Yang H. X., Zhu W. W., Yang H. Y., Li H. X., Kapur A. (2015). Effects of intervention to mild GDM on outcomes. *The Journal of Maternal-Fetal & Neonatal Medicine*.

[54] Li H. P., Wang F. H., Tao M. F., Huang Y. J., Jia W. P. (2016). Association between glycemic control and birthweight with glycated albumin in Chinese women with gestational diabetes mellitus. *Journal of Diabetes Investigation*.

[55] Wei Y. M., Yan J., Yang H. X. (2016). Identification of severe gestational diabetes mellitus after new criteria used in China. *Journal of Perinatology*.

[56] Xu Q., Gao Z. Y., Li L. M. (2016). The Association of Maternal Body Composition and Dietary Intake with the risk of gestational diabetes mellitus during the second trimester in a cohort of Chinese pregnant women. *Biomedical and Environmental Sciences*.

[57] Ye M., Liu Y., Cao X. (2016). The utility of HbA1c for screening gestational diabetes mellitus and its relationship with adverse pregnancy outcomes. *Diabetes Research and Clinical Practice*.

[58] Yang X., Hsu-Hage B., Zhang H. (2002). Gestational diabetes mellitus in women of single gravidity in Tianjin City, China. *Diabetes Care*.

[59] Fan Z., Yang H., Gao X., Lintu H., Sun W. (2006). Pregnancy outcome in gestational diabetes. *International Journal of Gynecology & Obstetrics*.

[60] Yang H., Wei Y., Gao X. (2009). Risk factors for gestational diabetes mellitus in Chinese women—a prospective study of 16 286 pregnant women in China. *Diabetic Medicine*.

[61] Wang Y., Nie M., Li W. (2011). Association of six single nucleotide polymorphisms with gestational diabetes mellitus in a Chinese population. *PLoS One*.

[62] Zhang F., Dong L., Zhang C. P. (2011). Increasing prevalence of gestational diabetes mellitus in Chinese women from 1999 to 2008. *Diabetic Medicine*.

[63] Chang Y., Chen X., Cui H., Zhang Z., Cheng L. (2014). Follow-up of postpartum women with gestational diabetes mellitus (GDM). *Diabetes Research and Clinical Practice*.

[64] Liu G., Li N., Sun S. (2014). Maternal OGTT glucose levels at 26–30 gestational weeks with offspring growth and development in early infancy. *BioMed Research International*.

[65] Jang H. C., Yim C. H., Han K. O. (2003). Gestational diabetes mellitus in Korea: prevalence and prediction of glucose intolerance at early postpartum. *Diabetes Research and Clinical Practice*.

[66] Lin C. H., Wen S. F., Wu Y. H., Huang M. J. (2009). Using the 100-g oral glucose tolerance test to predict fetal and maternal outcomes in women with gestational diabetes mellitus. *Chang Gung Medical Journal*.

[67] Chuang C. M., Lin I. F., Horng H. C., Hsiao Y. H., Shyu I. L., Chou P. (2012). The impact of gestational diabetes mellitus on postpartum urinary incontinence: a longitudinal cohort study on singleton pregnancies. *BJOG: An International Journal of Obstetrics and Gynaecology*.

[68] Park S., Kim M. Y., Baik S. H. (2013). Gestational diabetes is associated with high energy and saturated fat intakes and with low plasma visfatin and adiponectin levels independent of prepregnancy BMI. *European Journal of Clinical Nutrition*.

[69] Wang P., Lu M. C., Yu C. W., Wang L. C., Yan Y. H. (2013). Influence of food intake on the predictive value of the gestational diabetes mellitus screening test. *Obstetrics & Gynecology*.

[70] Yang S. J., Kim T. N., Baik S. H. (2013). Insulin secretion and insulin resistance in Korean women with gestational diabetes mellitus and impaired glucose tolerance. *The Korean Journal of Internal Medicine*.

[71] Cho G. J., Kim L. Y., Sung Y. N. (2015). Secular trends of gestational diabetes mellitus and changes in its risk factors. *PLoS One*.

[72] Heo J. M., Kim T. H., Hahn M. H. (2015). Comparison of the effects of gestational weight gain on pregnancy outcomes between non-diabetic and diabetic women. *Obstetrics & Gynecology Science*.

[73] Iimura Y., Matsuura M., Yao Z. (2015). Lack of predictive power of plasma lipids or lipoproteins for gestational diabetes mellitus in Japanese women. *Journal of Diabetes Investigation*.

[74] Jung Y. J., Kwon J. Y., Cho H. Y., Park Y. W., Kim Y. H. (2015). Comparison of the performance of screening test for gestational diabetes in singleton versus twin pregnancies. *Obstetrics & Gynecology Science*.

[75] Koo B. K., Lee J. H., Kim J., Jang E. J., Lee C.-H. (2016). Prevalence of gestational diabetes mellitus in Korea: a national health insurance database study. *PLoS One*.

[76] Ohara R., Obata-Yasuoka M., Abe K., Yagi H., Hamada H., Yoshikawa H. (2016). Effect of hyperemesis gravidarum on gestational diabetes mellitus screening. *International Journal of Gynecology & Obstetrics*.

[77] Park J. S., Kim D. W., Kwon J. Y., Park Y. W., Kim Y. H., Cho H. Y. (2016). Development of a screening tool for predicting adverse outcomes of gestational diabetes mellitus: a retrospective cohort study. *Medicine*.

[78] Shimodaira M., Yamasaki T., Nakayama T. (2016). The association of maternal ABO blood group with gestational diabetes mellitus in Japanese pregnant women. *Diabetes & Metabolic Syndrome: Clinical Research & Reviews*.

[79] Boriboonhirunsarn D., Sunsaneevithayakul P., Nuchangrid M. (2004). Incidence of gestational diabetes mellitus diagnosed before 20 weeks of gestation. *Journal of the Medical Association of Thailand*.

[80] Punthumapol C., Tekasakul P. (2008). 50 grams glucose challenge test for screening of gestational diabetes mellitus in each trimester in potential diabetic pregnancy. *Journal of the Medical Association of Thailand*.

[81] Ruangvutilert P., Chaemsaithong P., Ruangrongmorakot K., Kanokpongsakdi S., Sunsaneevithayakul P. (2010). Development of a modified 100-gram oral glucose tolerance test for diagnosis of gestational diabetes mellitus and its diagnostic accuracy. *Journal of the Medical Association of Thailand*.

[82] Tan P. C., Chai J. N., Ling L. P., Omar S. Z. (2011). Maternal hemoglobin level and red cell indices as predictors of gestational diabetes in a multi-ethnic Asian population. *Clinical and Experimental Obstetrics & Gynecology*.

[83] Srichumchit S., Luewan S., Tongsong T. (2015). Outcomes of pregnancy with gestational diabetes mellitus. *International Journal of Gynecology & Obstetrics*.

[84] DeSisto C. L., Kim S. Y., Sharma A. J. (2014). Prevalence estimates of gestational diabetes mellitus in the United States, pregnancy risk assessment monitoring system (PRAMS), 2007–2010. *Preventing Chronic Disease*.

[85] Chamberlain C., Joshy G., Li H., Oats J., Eades S., Banks E. (2015). The prevalence of gestational diabetes mellitus among aboriginal and Torres Strait Islander women in Australia: a systematic review and meta-analysis. *Diabetes/Metabolism Research and Reviews*.

[86] Eades C. E., Cameron D. M., Evans J. M. M. (2017). Prevalence of gestational diabetes mellitus in Europe: a meta-analysis. *Diabetes Research and Clinical Practice*.

[87] Yuen L., Wong V. W. (2015). Gestational diabetes mellitus: challenges for different ethnic groups. *World Journal of Diabetes*.

[88] Barker D. J. P. (2007). The origins of the developmental origins theory. *Journal of Internal Medicine*.

[89] National Diabetes Data Group (1979). Classification and diagnosis of diabetes mellitus and other categories of glucose intolerance. *Diabetes*.

[90] Carpenter M. W., Coustan D. R. (1982). Criteria for screening tests for gestational diabetes. *American Journal of Obstetrics and Gynecology*.

[91] American Diabetes Association (2004). Gestational diabetes mellitus. *Diabetes Care*.

[92] World Health Organization (1999). *Definition, Diagnosis and Classification of Diabetes Mellitus and its Complications. Part 1: Diagnosis and Classification of Diabetes Mellitus*.

[93] International Association of Diabetes and Pregnancy Study Groups Consensus Panel, Metzger B. E., Gabbe S. G. (2010). International association of diabetes and pregnancy study groups recommendations on the diagnosis and classification of hyperglycemia in pregnancy. *Diabetes Care*.

[94] Trujillo J., Vigo A., Duncan B. B. (2015). Impact of the International Association of Diabetes and Pregnancy Study Groups criteria for gestational diabetes. *Diabetes Research and Clinical Practice*.

[95] Hartling L., Dryden D. M., Guthrie A., Muise M., Vandermeer B., Donovan L. (2014). Diagnostic thresholds for gestational diabetes and their impact on pregnancy outcomes: a systematic review. *Diabetic Medicine*.

[96] International Association of Diabetes & Pregnancy Study Groups (IADPSG) Consensus Panel Writing Group and the Hyperglycemia & Adverse Pregnancy Outcome (HAPO) Study Steering Committee, Metzger B. E., Gabbe S. G. (2012). The diagnosis of gestational diabetes mellitus: new paradigms or status quo?. *The Journal of Maternal-Fetal & Neonatal Medicine*.

[97] Ko G. T. C., Chan J. C. N., Yeung V. T. F., Chow C.-C., Tsang L. W. W., Cockram C. S. (2001). A low socio-economic status is an additional risk factor for glucose intolerance in high risk Hong Kong Chinese. *European Journal of Epidemiology*.

[98] Bo S., Menato G., Bardelli C. (2002). Low socioeconomic status as a risk factor for gestational diabetes. *Diabetes & Metabolism*.

[99] Zhu Y., Zhang C. (2016). Prevalence of gestational diabetes and risk of progression to type 2 diabetes: a global perspective. *Current Diabetes Reports*.

