# Supplementary material for: Prevalence of Gestational Diabetes Mellitus in Eastern and Southeastern Asia: A Systematic Review and Meta-Analysis
Source: J Diabetes Res. 2018 Feb 20;2018:6536974. doi: 10.1155/2018/6536974 (PMC5838488; doi:10.1155/2018/6536974)
Supplement: Supplementary Materials — Supplemental Table: characteristics of selected studies. [file 6536974.f1.docx]

**Supplemental Table.** Characteristics of selected studies

| **First author, country, years data collected, year publication, reference** | **Setting** | **Gestation at testing** | **Steps** | **Sample size** | **GDM cases** | **Prevalence (95% CI)** | **Criteria used** |
| --- | --- | --- | --- | --- | --- | --- | --- |
| Li et al., China, 2010-2014, 2016 [54] | Shanghai Jiao Tong University Affiliated Sixth People's Hospital | 24-28 weeks | 2 steps: 50-g & 75-g | 2118 | 639 | 30.17 [28.22-32.17] | IADPSG 2010 (at least 1 criterion): Fasting glucose ≥5.1 mmol/l, 1h-OGTT ≥10.0 mmol/l, 2h-OGTT ≥8.5 mmol/l |
| Xu et al., China, 2012-2013, 2016 [56] | PLA General Hospital, Beijing | 24-28 weeks | NA | 1135 | 154 | 13.57 [11.63-15.70] | IADPSG 2010 (at least 1 criterion): Fasting glucose ≥5.1 mmol/l, 1h-OGTT ≥10.0 mmol/l, 2h-OGTT ≥8.5 mmol/l |
| Ye et al., China, 2012, 2016 [57] | First Affiliated Hospital of Sun Yat-sen University | 24-28 weeks | 1 step: 75-g | 1959 | 413 | 21.08 [19.29-22.96] | IADPSG 2010 (at least 1 criterion): Fasting glucose ≥5.1 mmol/l, 1h-OGTT ≥10.0 mmol/l, 2h-OGTT ≥8.5 mmol/l |
| Wei et al., China, 2011-2013, 2016 [55] | Peking University First Hospital | 24-28 weeks | 1 step: 75-g | 9803 | 2133 | 21.76 [20.95-22.59] | IADPSG 2010 (at least 1 criterion): Fasting glucose ≥5.1 mmol/l, 1h-OGTT ≥10.0 mmol/l, 2h-OGTT ≥8.5 mmol/l |
| Wei et al., China, 2005-2012, 2015 [53] | Peking University First Hospital | 24-28 weeks | Both 1 and 2 steps | 25674 | 3990 | 15.54 [15.10-15.99] | IADPSG 2010 (at least 1 criterion): Fasting glucose ≥5.1 mmol/l, 1h-OGTT ≥10.0 mmol/l, 2h-OGTT ≥8.5 mmol/l |
| He et al., China, 2012-2014, 2015 [49] | Guangzhou, China | NA | 1 step: 75-g | 3063 | 544 | 17.76 [16.42-19.13] | IADPSG 2010 (at least 1 criterion): Fasting glucose ≥5.1 mmol/l, 1h-OGTT ≥10.0 mmol/l, 2h-OGTT ≥8.5 mmol/l |
| Peng et al., China, 2012, 2015 [51] | Xiamen Maternity & Child Care Hospital, China | 24-28 weeks | 2 steps: 50-g & 75-g | 1359 | 166 | 12.21 [10.52-14.07] | WHO 1999: (1) diabetes as FPG ≥ 7.0 mmol/l and/or 2h-75g OGTT ≥11.1 mmol/l; (2) IGT as FPG <7.0 mmol/l and 2h-75g OGTT ≥7.8 mmol/l but <11.1 mmol/l |
| Li et al., China, 2013, 2015 [50] | Beijing Obsterics & Gynecology Hospital | 24-28 weeks | 1 step: 75-g | 2545 | 379 | 14.89 [13.53-16.34] | ADA2012 (at least 1 criterion): Fasting glucose ≥5.1 mmol/l, 1h-OGTT ≥10.0 mmol/l, 2h-OGTT ≥8.5 mmol/l |
| Wang et al., China, 2013, 2015 [52] | 15 hospitals in Beijing | ≥24 weeks | 1 step: 75-g | 14168 | 2750 | 19.41 [18.76-20.07] | IADPSG 2010 (at least 1 criterion): Fasting glucose ≥5.1 mmol/l, 1h-OGTT ≥10.0 mmol/l, 2h-OGTT ≥8.5 mmol/l |
| Zhu et al., China, 2010-2012, 2015 [48] | 13 hospitals including Peking University First Hospital | 24-28 weeks | 1 step: 75-g | 17186 | 3002 | 17.47 [16.90-18.04] | WHO 2013, 75g OGTT (at least 1 criterion): Fasting glucose ≥5.1 mmol/l, 1h-OGTT ≥10.0 mmol/l, 2h-OGTT ≥8.5 mmol/l |
| Zhu et al., China, 2010-2012, 2015 [48] | 13 hospitals including Peking University First Hospital | 24-28 weeks | 1 step: 75-g | 17186 | 2952 | 17.18 [16.62-17.75] | WHO 1999: Fasting glucose ≥7.0 mmol/L or 2 hours ≥ 7.8 mmol/L |
| Leng et al., China, 2010-2012, 2015 [45] | Six central urban districts in Tianjin | 24-28 weeks | 2 steps: 50-g & 75-g | 18589 | 1721 | 9.26 [8.85-9.68] | IADPSG 2010 (at least 1 criterion): Fasting glucose ≥5.1 mmol/l, 1h-OGTT ≥10.0 mmol/l, 2h-OGTT ≥8.5 mmol/l |
| Leng et al., China, 2010-2012, 2015 [45] | Six central urban districts in Tianjin | 24-28 weeks | 2 steps: 50-g & 75-g | 18589 | 1506 | 8.10 [7.71-8.50] | WHO 1999: (1) diabetes as FPG ≥7.0 mmol/l and/or 2h-75g OGTT ≥11.1 mmol/l; (2) IGT as FPG <7.0 mmol/l and 2h-75g OGTT ≥7.8 mmol/l but <11.1 mmol/l; (3) IFG as FPG ≥6.1 mmol/l but <7.0 mmol/l and 2h-75g OGTT <7.8 mmol/l |
| Shang et al., China, 2012-2013, 2014 [42] | Beijing Friendship Hospital | 24-28 weeks | 1 step: 75-g | 3083 | 246 | 7.98 [7.05-8.99] | ADA 2010, 75g OGTT (at least 2 criteria): Fasting glucose ≥5.5 mg/dL, 1h-OGTT ≥180 mg/dL, 2h-OGTT ≥155 mg/dL |
| Shang et al., China, 2012-2013, 2014 [42] | Beijing Friendship Hospital | 24-28 weeks | 1 step: 75-g | 3083 | 612 | 19.85 [18.46-21.30] | IADPSG 2010 (at least 1 criterion): Fasting glucose ≥92 mg/dL, 1h-OGTT ≥180 mg/dL, 2h-OGTT ≥153 mg/dL |
| Shang et al., China, 2008-2011, 2014 [43] | Beijing Friendship Hospital | 24-28 weeks | 2 steps: 50-g & 75-g | 6201 | 570 | 9.19 [8.48-9.94] | ADA 2010, 75g OGTT (at least 2 criteria): Fasting glucose ≥5.3 mmol/l, 1h-OGTT ≥10.0 mmol/l, 2h-OGTT ≥8.6 mmol/l |
| Shang et al., China, 2008-2011, 2014 [43] | Beijing Friendship Hospital | 24-28 weeks | 2 steps: 50-g & 75-g | 6201 | 676 | 10.90 [10.14-11.70] | IADPSG 2010 (at least 1 criterion): Fasting glucose ≥5.1 mmol/l, 1h-OGTT ≥10.0 mmol/l, 2h-OGTT ≥8.5 mmol/l |
| Liao et al., China, 2008-2011, 2014 [41] | Sichuan Provincial People's Hospital | 24-28 weeks | 2 steps: 50-g & 100-g | 5360 | 626 | 11.68 [10.83-12.57] | ADA2007 (at least 2 criteria), 100g OGTT: Fasting ≥5.3 mmol/l, 1h-OGTT ≥10.0 mmol/l, 2h-OGTT ≥ 8.6 mmol/l, 3h-OGTT ≥ 7.8 mmol/l |
| Liao et al., China, 2008-2011, 2014 [41] | Sichuan Provincial People's Hospital | 24-28 weeks | 2 steps: 50-g & 100-g | 5360 | 1314 | 24.51 [23.37-25.69] | IADPSG 2010 (at least 1 criterion): Fasting glucose ≥5.1 mmol/l, 1h-OGTT ≥10.0 mmol/l, 2h-OGTT ≥8.5 mmol/l |
| Liu et al., China, 2009-2011, 2014 [64] | Six central urban districts in Tianjin | 26-30 weeks | 2 steps: 50-g & 75-g | 27157 | 1420 | 5.23 [4.97-5.50] | WHO 2006, 75g OGTT: IFG ≥7.0 mmol/L; IGT: FG <7.0 mmol/L and 2h-OGTT ≥7.8 - <11.1 mmol/L; newly diagnosed diabetes: GCT ≥7.8 and FG ≥7.0 or 2h ≥11.1 mmol/L. |
| Chang et al., China, 2008-2010, 2014 [63] | Tianjin Center Hospital of Obstetrics and Gynecology | 24-28 weeks | 1 step: 75-g | 10852 | 1010 | 9.31 [8.77-9.87] | ADA2007 (at least 2 criteria): (1) 100g OGTT: Fasting ≥5.3 mmol/l, 1h-OGTT ≥10.0 mmol/l, 2h-OGTT ≥8.6 mmol/l, 3h-OGTT ≥7.8 mmol/l; (2) 75g OGTT: Fasting ≥5.3 mmol/l, 1h-OGTT ≥10.0 mmol/l, 2h-OGTT ≥8.6 mmol/l |
| Zhang et al., China, 1999-2008, 2011 [62] | Six central urban districts in Tianjin | 26-30 weeks | 2 steps: 50-g & 75-g | 105473 | 5185 | 4.92 [4.79-5.05] | WHO 1999: (1) diabetes as FPG ≥7.0 mmol/l and/or 2h-75g OGTT ≥11.1 mmol/l; (2) IGT as FPG <7.0 mmol/l and 2h-75g OGTT ≥7.8 mmol/l but <11.1 mmol/l; (3) IFG as FPG ≥6.1 mmol/l but <7.0 mmol/l and 2h-75g OGTT <7.8 mmol/l |
| Wang et al., China, 2006-2010, 2011 [61] | Peking Union Medical College Hospital | 24-28 weeks | 2 steps: 50-g & 100-g | 1764 | 725 | 41.10 [38.79-43.44] | ADA2007 (at least 2 criteria), 100g OGTT: Fasting ≥5.3 mmol/l, 1h-OGTT ≥10.0 mmol/l, 2h-OGTT ≥8.6 mmol/l, 3h-OGTT ≥7.8 mmol/l |
| Yang et al., China, 2006, 2009 [60] | 26 hospitals in 18 cities | 24-28 weeks | 2 steps: 50-g & 75-g | 16286 | 708 | 4.35 [4.04-4.67] | ADA 2010, 75g OGTT (at least 2 criteria): Fasting glucose ≥5.3 mmol/l, 1h-OGTT ≥10.0 mmol/l, 2h-OGTT ≥8.6 mmol/l |
| Fan et al., China, 1995-2004, 2006 [59] | Peking University First Hospital | 24-28 weeks | 2 steps: 50-g & 75-g | 20512 | 782 | 3.81 [3.55-4.08] | NDDG (Modified): diagnosis as fasting >5.8 mmol/l or ≥2 values: 1h-OGTT ≥10.6 mmol/l, 2h-OGTT ≥ 9.2 mmol/l, 3h-OGTT ≥8.1 mmol/l |
| Yang et al., China, 1998-1999, 2002 [58] | Six central urban districts in Tianjin | 26-30 weeks | 2 steps: 50-g & 75-g | 9471 | 219 | 2.31 [2.02-2.64] | WHO 1998, 75g OGTT: Fasting glucose ≥ 7.0 mmol/L and/or 2h-OGTT ≥11.1 mmol/L and Impaired glucose tolerance: FG <7.0 mmol/L and 2h-OGTT ≥7.8-11.1 mmol/L |
| Hung et al., Taiwan, 2009-2010, 2015 [44] | Chang Gung Memorial Hospital, Taipei | 24-28 weeks | 2 steps: 50-g & 100-g | 3056 | 141 | 4.61 [3.90-5.42] | Carpenter-Coustan, 100g OGTT (at least 2 criteria): Fasting glucose ≥95 mg/dL, 1h-OGTT ≥180 mg/dL, 2h-OGTT ≥155 mg/dL, 3h-OGTT ≥140 mg/dL |
| Hung et al., Taiwan, 2012-2013, 2015 [44] | Chang Gung Memorial Hospital, Taipei | 24-28 weeks | 1 step: 75-g | 3641 | 453 | 12.44 [11.39-13.56] | IADPSG 2010 (at least 1 criterion): Fasting glucose ≥92 mg/dL, 1h-OGTT ≥180 mg/dL, 2h-OGTT ≥153 mg/dL |
| Wang et al., Taiwan, 2011, 2013 [69] | Chia-Yi Christian Hospital, Taiwan | 24-28 weeks | 2 steps: 50-g & 100-g | 1387 | 60 | 4.33 [3.32-5.53] | NDDG, 100g OGTT (at least 2 criteria): Fasting glucose ≥105 mg/dL, 1h-OGTT ≥190 mg/dL, 2h-OGTT ≥165 mg/dL, 3h-OGTT ≥145 mg/dL |
| Chuang CM, Taiwan, 2002-2007, 2012 [67] | Tertiary hospital | 24-28 weeks | 2 steps: 50-g & 100-g | 6369 | 562 | 8.82 [8.14-9.55] | NDDG, 100g OGTT (at least 2 criteria): Fasting >5.8 mmol/l; 1h-OGTT ≥ 10.5 mmol/l; 2h-OGTT ≥ 9.2 mmol/l; 3h-OGTT ≥8.0 mmol/l |
| Chou et al., Taiwan, 2001-2008, 2010 [38] | Cathay General Hospital, Taiwan | 24-28 weeks | 2 steps: 50-g & 100-g | 10990 | 489 | 4.45 [4.07-4.85] | Carpenter-Coustan, 100g OGTT (at least 2 criteria): Fasting glucose ≥95 mg/dL, 1h-OGTT ≥180 mg/dL, 2h-OGTT ≥155 mg/dL, 3h-OGTT ≥140 mg/dL |
| Chou et al., Taiwan, 2001-2008, 2010 [38] | Cathay General Hospital, Taiwan | 24-28 weeks | 2 steps: 50-g & 100-g | 10990 | 385 | 3.50 [3.17-3.86] | NDDG, 100g OGTT (at least 2 criteria): Fasting glucose ≥105 mg/dL, 1h-OGTT ≥190 mg/dL, 2h-OGTT ≥165 mg/dL, 3h-OGTT ≥145 mg/dL |
| Lin et al., Taiwan, 2001-2006, 2009 [66] | NA | 24-28 weeks | 2 steps: 50-g & 100-g | 8557 | 636 | 7.43 [6.89-8.01] | Carpenter-Coustan, 100g OGTT (at least 2 criteria): Fasting glucose ≥95 mg/dL, 1h-OGTT ≥180 mg/dL, 2h-OGTT ≥155 mg/dL, 3h-OGTT ≥140 mg/dL |
| Shimodaira et al., Japan, 2010-2015, 2016 [78] | Iida Municipal Hospital | NA | 2 steps: 50-g & 100-g | 5424 | 149 | 2.75 [2.33-3.22] | IADPSG 2010 (at least 1 criterion): Fasting glucose ≥92 mg/dL, 1h-OGTT ≥180 mg/dL, 2h-OGTT ≥153 mg/dL |
| Ohara et al., Japan, 2010-2013, 2016 [76] | Tsukuba University Hospital | 1st and 2nd trimesters | 2 steps: 50-g & 75-g | 2112 | 275 | 13.02 [11.61-14.53] | IADPSG 2010 (at least 1 criterion): Fasting glucose ≥5.1 mmol/l, 1h-OGTT ≥10.0 mmol/l, 2h-OGTT ≥8.5 mmol/l |
| Limura et al., Japan, 2010-2011, 2015 [73] | Japanese Red Cross Medical Centre | 20-28 weeks | 2 steps: 50-g & 75-g | 1183 | 53 | 4.48 [3.37-5.82] | IADPSG 2010 (at least 1 criterion): Fasting glucose ≥92 mg/dL, 1h-OGTT ≥180 mg/dL, 2h-OGTT ≥153 mg/dL |
| Nobumoto et al., Japan, 2001-2011, 2015 [47] | 28 hospitals in Japan | ≤28 weeks | 1 step: 75-g | 2839 | 83 | 2.92 [2.34-3.61] | Japan Society of Obstetrics and Gynecology (at least 2 criteria): Fasting glucose ≥ 100 mg/dL, 1h-OGTT ≥180 mg/dL, 2h-OGTT ≥150 mg/dL |
| Nobumoto et al., Japan, 2001-2011, 2015 [47] | 28 hospitals in Japan | ≤28 weeks | 1 step: 75-g | 2839 | 363 | 12.79 [11.58-14.07] | IADPSG 2010 (at least 1 criterion): Fasting glucose ≥92 mg/dL, 1h-OGTT ≥180 mg/dL, 2h-OGTT ≥153 mg/dL |
| Morikawa et al., Japan, 2002-2006, 2010 [39] | Hokkaido University, Sapporo | 24-28 weeks | 2 steps: 50-g & 75-g | 1038 | 25 | 2.41 [1.56-3.53] | Japan Society of Obstetrics and Gynecology (at least 2 criteria): Fasting glucose ≥100 mg/dL, 1h-OGTT ≥180 mg/dL, 2h-OGTT ≥150 mg/dL |
| Morikawa et al., Japan, 2002-2006, 2010 [39] | Hokkaido University, Sapporo | 24-28 weeks | 2 steps: 50-g & 75-g | 1038 | 68 | 6.55 [5.12-8.23] | IADPSG 2010 (at least 1 criterion): Fasting glucose ≥5.1 mmol/l, 1h-OGTT ≥10.0 mmol/l, 2h-OGTT ≥8.5 mmol/l |
| Koo et al., Korea, 2009-2011, 2016 [75] | Health Insurance Review and Assessment database | NA | NA | 1,306,281 | 98,403 | 7.53 [7.51-7.56] | ICD10 code O244 |
| Park et al., Korea, 2001-2013, 2016 [77] | Severance Hospital, Seoul | ≥ 24 weeks | 2 steps: 50-g & 100-g | 3,434 | 306 | 8.91 [7.98-9.91] | Carpenter-Coustan, 100g OGTT (at least 2 criteria): Fasting glucose ≥95 mg/dL, 1h-OGTT ≥180 mg/dL, 2h-OGTT ≥155 mg/dL, 3h-OGTT ≥140 mg/dL |
| Cho et al., Korea, 2007-2010, 2015 [71] | Health Insurance Review and Assessment database | 24-28 weeks | NA | 1,824,913 | 129,666 | 7.11 [7.08-7.13] | Carpenter-Coustan, 100g OGTT (at least 2 criteria): Fasting glucose ≥95 mg/dL, 1h-OGTT ≥180 mg/dL, 2h-OGTT ≥155 mg/dL, 3h-OGTT ≥140 mg/dL |
| Heo et al., Korea, 2009-2013, 2015 [72] | Korea University Medical Center | NA | 2 steps: 50-g & 100-g | 5,212 | 322 | 6.18 [5.54-6.87] | Carpenter-Coustan, 100g OGTT (at least 2 criteria): Fasting glucose ≥95 mg/dL, 1h-OGTT ≥180 mg/dL, 2h-OGTT ≥155 mg/dL, 3h-OGTT ≥140 mg/dL |
| Jung YJ, Korea, 2000-2008, 2015 [74] | Severance Hospital, Seoul | 24-28 weeks | 2 steps: 50-g & 100-g | 3,435 | 286 | 8.33 [7.42-9.30] | Carpenter-Coustan, 100g OGTT (at least 2 criteria): Fasting glucose ≥95 mg/dL, 1h-OGTT ≥180 mg/dL, 2h-OGTT ≥155 mg/dL, 3h-OGTT ≥140 mg/dL |
| Park et al., Korea, 2006-2010, 2013 [68] | Cheil General Hospital & Women's Healthcare Center, Seoul | 24-28 weeks | 2 steps: 50-g & 100-g | 19,423 | 1,086 | 5.59 [5.27-5.92] | Carpenter-Coustan, 100g OGTT (at least 2 criteria): Fasting glucose ≥5.3 mM, 1h-OGTT ≥10 mM, 2h-OGTT ≥8.6 mM, 3h-OGTT ≥7.8 mM |
| Yang et al., Korea, 2004-2006, 2013 [70] | Cheil General Hospital & Women's Healthcare Center, Seoul | 24-28 weeks | 2 steps: 50-g & 100-g | 1,163 | 269 | 23.13 [20.73-25.66] | Carpenter-Coustan, 100g OGTT (at least 2 criteria): Fasting glucose ≥5.3 mM, 1h-OGTT ≥10 mM, 2h-OGTT ≥8.6 mM, 3h-OGTT ≥7.8 mM |
| Jang et al., Korea, 1993-1997, 2003 [65] | Samsung Cheil Hospital, Seoul | 24-28 weeks | 2 steps: 50-g & 100-g | 16654 | 392 | 2.35 [2.13-2.60] | NDDG, 100g OGTT (at least 2 criteria): Fasting >5.8 mmol/l; 1h-OGTT ≥10.6 mmol/l; 2h-OGTT ≥9.2 mmol/l; 3h-OGTT ≥8.1 mmol/l |
| Tan et al., Malaysia, NA, 2011 [82] | A university hospital in Kuala Lumpur | NA | 2 steps: 50-g & 75-g | 1538 | 182 | 11.83 [10.26-13.55] | WHO 1999 (at least 1 criteria): Fasting glucose ≥7.0 mmol/l, 2h-OGTT ≥11.1 mmol/l |
| Chong et al., Singapore, 2009-2010, 2014 [40] | KK's Women and Children's Hospital; National University Hospital | 26-28 weeks | 1 step: 75-g | 1136 | 215 | 18.93 [16.69-21.33] | WHO 1999, 75g OGTT (at least 1 criteria): Fasting glucose ≥7.0 mmol/L, 2h-OGTT ≥ 7.8 mmol/L |
| Chong et al., Singapore, 2009-2010, 2014 [40] | KK's Women and Children's Hospital; National University Hospital | 26-28 weeks | 1 step: 75-g | 1136 | 111 | 9.77 [8.11-11.65] | WHO 1999, 75g OGTT (at least 1 criteria): Fasting glucose ≥7.0 mmol/L, 2h-OGTT ≥ 7.8 mmol/L |
| Luengmettakul et al., Thailand, 2012, 2015 [46] | Siriraj Hospital, Bangkok | 24-28 weeks | 2 steps: 50-g & 100-g | 3283 | 144 | 4.39 [3.71-5.14] | Carpenter-Coustan, 100g OGTT (at least 2 criteria): Fasting glucose ≥95 mg/dL, 1h-OGTT ≥180 mg/dL, 2h-OGTT ≥155 mg/dL, 3h-OGTT ≥140 mg/dL |
| Luengmettakul et al., Thailand, 2009-2011, 2015 [46] | Siriraj Hospital, Bangkok | 24-28 weeks | 2 steps: 50-g & 100-g | 10603 | 1047 | 9.87 [9.31-10.46] | NDDG, 100g OGTT (at least 2 criteria): Fasting glucose ≥105 mg/dL, 1h-OGTT ≥190 mg/dL, 2h-OGTT ≥165 mg/dL, 3h-OGTT ≥145 mg/dL |
| Srichumchit et al., Thailand, 2002-2012, 2015 [83] | Maharaj Nakorn Chiang Mai Hospital | 24-28 weeks | 2 steps: 50-g & 100-g | 25255 | 1350 | 5.35 [5.07-5.63] | NDDG, 100g OGTT (at least 2 criteria): Fasting glucose ≥105 mg/dL, 1h-OGTT ≥190 mg/dL, 2h-OGTT ≥165 mg/dL, 3h-OGTT ≥145 mg/dL |
| Ruangvutilert et al., Thailand, 2005-2006, 2010 [81] | Siriraj Hospital, Bangkok | 24-34 weeks | 2 steps: 50-g & 100-g | 6812 | 403 | 5.92 [5.37-6.50] | NDDG, 100g OGTT (at least 2 criteria): Fasting glucose ≥105 mg/dL, 1h-OGTT ≥190 mg/dL, 2h-OGTT ≥165 mg/dL, 3h-OGTT ≥145 mg/dL |
| Punthumapol et al., Thailand, 2004-2005, 2008 [80] | Taksin Hospital, Bangkok | All trimesters | 2 steps: 50-g & 100-g | 2010 | 147 | 7.31 [6.21-8.54] | NDDG, 100g OGTT (at least 2 criteria): Fasting glucose ≥105 mg/dL, 1h-OGTT ≥190 mg/dL, 2h-OGTT ≥165 mg/dL, 3h-OGTT ≥145 mg/dL |
| Boriboonhirunsarn et al., Thailand, 2001, 2004 [79] | Siriraj Hospital, Bangkok | <20 weeks | 2 steps: 50-g & 100-g | 1200 | 119 | 9.92 [8.28-11.75] | NDDG, 100g OGTT (at least 2 criteria): Fasting glucose ≥105 mg/dL, 1h-OGTT ≥190 mg/dL, 2h-OGTT ≥165 mg/dL, 3h-OGTT ≥145 mg/dL |
| Sunsaneevithayakul P, Thailand, 2000, 2003 [37] | Siriraj Hospital, Bangkok | ≤32 weeks | 2 steps: 50-g & 100-g | 3770 | 235 | 6.23 [5.48-7.05] | NDDG, 100g OGTT (at least 2 criteria): Fasting glucose ≥105 mg/dL, 1h-OGTT ≥190 mg/dL, 2h-OGTT ≥165 mg/dL, 3h-OGTT ≥145 mg/dL |
| Sunsaneevithayakul P, Thailand, 2000, 2003 [37] | Siriraj Hospital, Bangkok | ≤32 weeks | 2 steps: 50-g & 100-g | 9325 | 235 | 2.52 [2.21-2.86] | NDDG, 100g OGTT (at least 2 criteria): Fasting glucose ≥105 mg/dL, 1h-OGTT ≥190 mg/dL, 2h-OGTT ≥165 mg/dL, 3h-OGTT ≥145 mg/dL |
| Tran et al., Viet Nam, 2010-2011, 2013 [36] | Hung Vuong hospital, HCM city | 24-32 weeks | 1 step: 75-g | 2772 | 164 | 5.92 [5.07-6.86] | ADA 2010, 75g OGTT (at least 2 criteria): Fasting glucose ≥5.3 mmol/l, 1h-OGTT ≥10.0 mmol/l, 2h-OGTT ≥8.6 mmol/l |
| Tran et al., Viet Nam, 2010-2011, 2013 [36] | Hung Vuong hospital, HCM city | 24-32 weeks | 1 step: 75-g | 2772 | 565 | 20.38 [18.90-21.93] | IADPSG 2010 (at least 1 criteria): Fasting glucose ≥5.1 mmol/l, 1h-OGTT ≥10.0 mmol/l, 2h-OGTT ≥8.5 mmol/l |
| Tran et al., Viet Nam, 2010-2011, 2013[36] | Hung Vuong hospital, HCM city | 24-32 weeks | 1 step: 75-g | 2772 | 577 | 20.82 [19.32-22.38] | ADIPS 1998 (at least 1 criteria): Fasting glucose ≥5.5 mmol/l, 2h-OGTT ≥8.0 mmol/l |
| Tran et al., Viet Nam, 2010-2011, 2013 [36] | Hung Vuong hospital, HCM city | 24-32 weeks | 1 step: 75-g | 2772 | 674 | 24.31 [22.73-25.96] | WHO 1999 (at least 1 criteria): Fasting glucose ≥7.0 mmol/l, 2h-OGTT ≥11.1 mmol/l |

**Abbreviations**: CI: confidence interval; NA: not available; OGTT: oral glucose tolerance test; ADA: American Diabetes Association; ADIPS: Australian Diabetes in Pregnancy Society; CC: Carpenter-Coustan; IADPSG: International Association of the Diabetes and Pregnancy Study Groups; ICD: International Classification of Diseases; JSOG: Japan Society of Obstetrics and Gynecology; NDDG: National Diabetes Data Group; WHO: World Health Organisation.
